# Chronic Hepatitis C Virus Infection Is Associated with the Development of Rheumatoid Arthritis: A Nationwide Population-Based Study in Taiwan

**DOI:** 10.1371/journal.pone.0113579

**Published:** 2014-11-21

**Authors:** Fu-Hsiung Su, Chien-Sheng Wu, Fung-Chang Sung, Shih-Ni Chang, Chien-Tien Su, Ying-Hua Shieh, Chih-Ching Yeh

**Affiliations:** 1 Department of Family Medicine, Taipei Medical University Hospital, Taipei, Taiwan; 2 Department of Family Medicine, School of Medicine, Taipei Medical University, Taipei, Taiwan; 3 Division of Allergy, Immunology and Rheumatology, Far Eastern Memorial Hospital, Taipei, Taiwan; 4 Graduate Institute of Clinical Medical Science, School of Medicine, College of Medicine, China Medical University, Taichung, Taiwan; 5 Management Office for Health Data, China Medical University Hospital, Taichung, Taiwan; 6 School of Public Health, College of Public Health and Nutrition, Taipei Medical University, Taipei, Taiwan; 7 Department of Family Medicine, Taipei Medical University, Wan Fang Hospital, Taipei, Taiwan; 8 Department of Public Health, China Medical University, Taichung, Taiwan; Centers for Disease Control and Prevention, United States of America

## Abstract

**Objective:**

The association between chronic hepatitis virus infection and rheumatoid arthritis (RA) remains debatable. This nationwide population-based cohort study assessed the risk of RA among patients with a chronic infection of hepatitis B and/or C virus.

**Materials and Methods:**

We used data extracted from the claims of 1,000,000 randomly sampled individuals covered under the Taiwan National Health Insurance program. Among the 49,892 persons identified in 2000–2010 with chronic hepatitis virus infection, 35,652 had chronic HBV infection alone, 10,253 had chronic HCV infection alone, and 3,987 had chronic HBV/HCV dual infections. The comparison cohort comprised 199,568 persons matched on sex, age and calendar year without chronic hepatitis virus infection. All study participants were free of RA at baseline and traced through 2011 with new RA cases identified.

**Results:**

After adjusting for covariates, chronic HCV infection alone was significantly associated with an increased risk for RA (hazard ratio (HR)  = 2.03, 95% confidence interval (CI)  = 1.27–3.22). The increased risk for RA among participants with chronic HCV infection remained significant after restricting the analysis to those who were prescribed disease-modifying anti-rheumatic drugs. The corresponding HR for the overall sample was 1.89 (95% CI  = 1.15–3.11). However, HBV carriers did not appear to be at a significantly higher risk for RA.

**Conclusion:**

Our data imply that chronic HCV infection is associated with RA development.

## Introduction

Hepatitis B (HBV) and hepatitis C (HCV) virus infections are important global health concerns [1]. It has been estimated that around 350 million people worldwide are chronic HBV carriers with one million people dying annually from HBV-related diseases such as acute hepatitis/chronic hepatitis, cirrhosis of the liver, and hepatocellular carcinoma (HCC) [2]. The estimated global prevalence of HCV infection is 2.2%, corresponding to about 130 million HCV-positive persons worldwide [3]. Overall, 75–80% of global HCC cases are attributable to persistent viral infections with either HBV (50–55%) or HCV (25–30%) [4]. In 2007, Taiwan launched a series of community-based screening programs for liver diseases. The results of this screening estimated a 17.3% seroprevalence of HBV and a 4.4% seroprevalence of HCV in the general population aged ≥18 years [5]. In Taiwan, chronic HBV infection has the strongest association with the development of HCC and accounts for 80% of all HCC cases, while HCV infection accounts for approximately 15% of Taiwan's HCC patients [6]. Thus, chronic HBV and HCV infections remain extremely important health problems in Taiwan.

Besides hepatic manifestations, chronic HBV and HCV infections have many extra-hepatic manifestations [7],[8]. Extra-hepatic manifestations such as arthropathy are clinically evident in about 40–70% of patients with HCV infection [9], while are present in up to 20% of HBV-infected patients [8]. Rheumatologic involvement is the most frequent extra-hepatic manifestation of both HCV and HBV infections and has therefore attracted increasing interest [10],[11]. In a large prospective study of 1,614 patients with chronic HCV infection, 23% of the patients were reported to generically suffer from arthralgia and arthritis [11]. Two further studies have suggested a high seroprevalence of HCV infection among patients with rheumatic diseases [12],[13]. Previous studies also suggested that chronic HBV infection may act as a trigger for the development of autoimmune rheumatic diseases (ARDs) [14],[15] and observed a high positive rate of rheumatoid factor (RF) among asymptomatic HBV carriers [16],[17]. However, other studies have failed to detect a relationship between HBV infection and more common ARDs, including rheumatoid arthritis (RA) [18],[19].

In Taiwan, a nationwide population-based study reported the prevalence of RA to be 52.4 per 100,000 people [20]. Without proper treatment, about half of these RA patients may lose their working capacity within ten years, making RA an important public health concern [21]. The NHI database has been proven to be a useful tool in revealing possible interactions between clinical risk factors and RA [22]. Considering the high prevalence of chronic HBV and HCV infections in Taiwan, we have a unique opportunity to clarify the debate over whether chronic hepatitis virus infection is associated with the development of RA. We set out to investigate this putative association utilizing a nationwide population-based dataset of insurance claims.

## Materials and Methods

### Data sources

The data sourced for analysis in this study were retrieved from the Taiwan National Health Insurance Research Database (NHIRD). This database is managed by the Taiwan National Health Research Institutes (NHRI), Department of Health. The database includes the data from the state-run National Health Insurance (NHI) program and was initiated in 1995 to provide affordable health care for the island's 23 million inhabitants [23]. This compulsory-enrollment and single-payer insurance program covered approximately 99% of Taiwan's population of more than 23 million by the end of 2004 [24]. The Taiwan NHIRD contains the claims data of 1,000,000 individuals randomly selected from all insured enrollees. This sample represents the original medical claims for all islanders under the NHI program. We received approval to use the data for ambulatory care claims, inpatient claims, and updated registries from the year 2000 to the year 2011. Diagnoses were coded according to the International Classification of Disease, Ninth revision (ICD-9-CM). In Taiwan, patients diagnosed with RA are entitled to Catastrophic Illness Certificates, which are reviewed by rheumatologists commissioned by the Bureau of NHI. Therefore, the catastrophic illness patient data are highly accurate and reliable. The database used in this study can be interlinked by the scrambled unique individual personal identification numbers provided by the NHI. The surrogate identifications safeguard the privacy and confidentiality of all beneficiaries. This study was exempted from full ethical review by the International Review Board, China Medical University and Hospital Research Ethics Committee (IRB permit number: CMU-REC-101-012).

### Study sample

**Figure 1:**
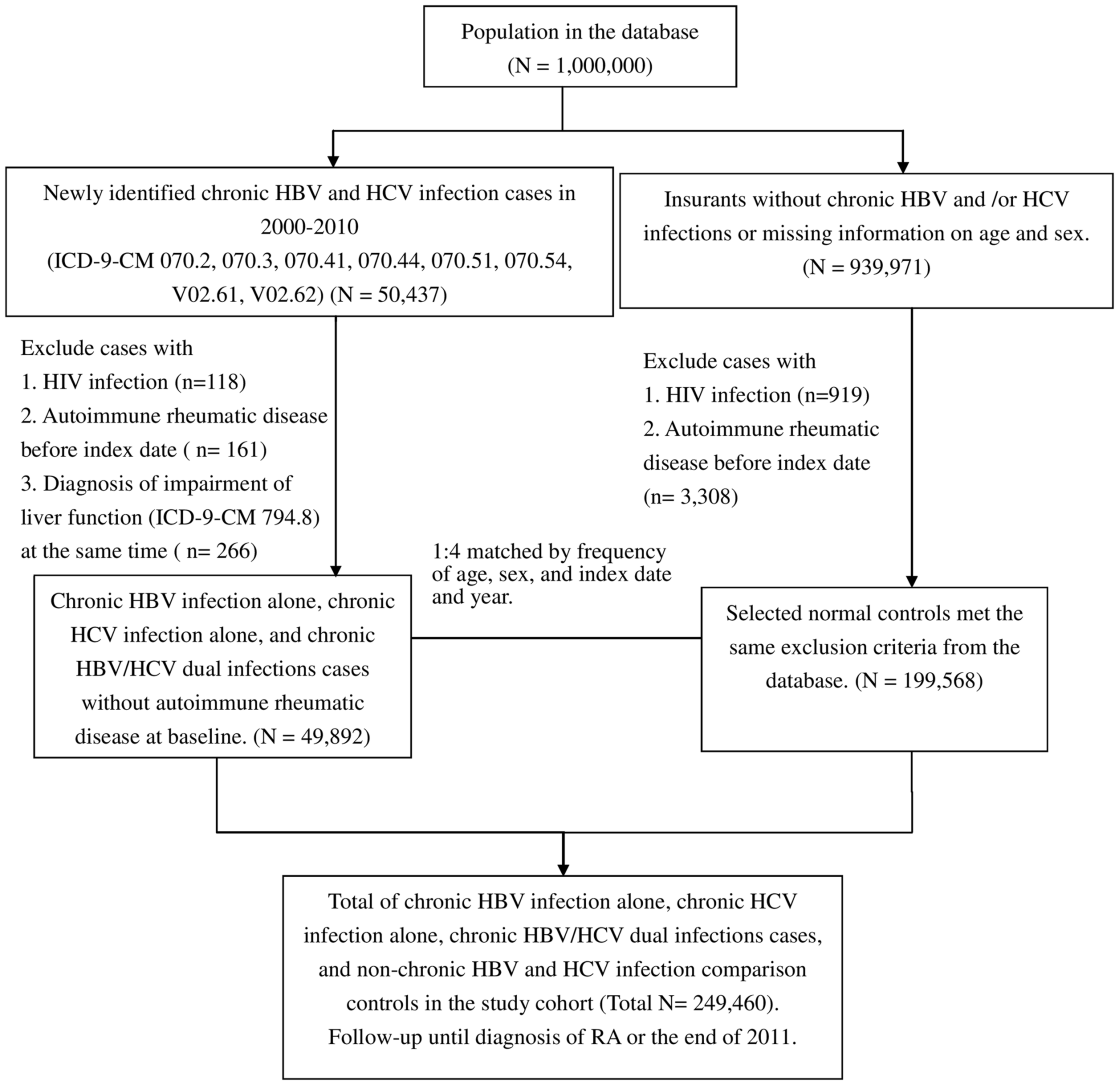
Study participant selection.

In this study, we identified patients with diagnoses of HBV (ICD-9-CM: 070.2, 070.3, V02.61) and HCV (ICD-9-CM: 070.41, 070.44, 070.51, 070.54, V02.62) infections during the period of 2000-2010. We excluded those with only one diagnosis of acute or unspecified HBV infection (ICD-9-CM: 070.20, 070.21, 0.70.30, 070.31) and those with only one diagnosis of acute or unspecified HCV infection (ICD-9-CM: 0.70.41, 0.70.51) concurrent with a diagnosis of impairment of liver function (ICD-9-CM: 794.8). Patients who received a second diagnosis of hepatitis virus infection six months after being diagnosed with acute or unspecified HBV or HCV infection were classified as chronic cases. The index date for patients with chronic HBV and HCV infections was the first instance of having chronic HCV or HBV infection identified. Participants with a diagnosis of autoimmune rheumatic diseases (ARDs) before the index date and subjects with a diagnosis of HIV infection at any time during the study period (ICD-9-CM: 042, 043, 044, V08 and 795.8) were excluded. ARDs were identified with codes 714.0 for RA, 710.0 for systemic lupus erythematosus, 710.2 for Sjögren's syndrome, 710.1 for progressive systemic sclerosis, 710.3 for polymyositis, 710.4 for dermatomyositis, 446.0–446.7 for vasculitis (including polyarteritis nodosa, Kawasaki disease, hypersensitivity angiitis, Wegener's granulomatosis, giant cell arteritis, and Takayasu's disease), and 136.1 for Behçet's disease. After excluding 161 cases of ARDs, 118 cases of HIV infection, and 266 cases of acute HBV or HCV infection, 35,652, 10,253, and 3,987 subjects with chronic HBV infection alone, chronic HCV infection alone, and chronic HBV/HCV dual infections were enrolled in this study and followed until having received a diagnosis of RA (ICD-9-CM: 714.0) or reaching the end of the year 2011 (Figure 1).

The non-chronic HBV and HCV infection comparison group was composed of the remaining patients in the NHI database who did not receive any ICD-9-CM diagnostic codes for hepatitis virus infection (ICD-9-CM: 070.X), chronic hepatitis (ICD-9-CM: 571.4, 571.8, 571.9, and 573.3), or carriers or suspected carriers of hepatitis virus infection (ICD-9-CM: V02.6). Among the 939,971 participants without HBV and/or HCV infections, after excluding 3,308 cases of ARDs and 919 cases of HIV infection, in total of 935,744 participants remained eligible for the non-chronic HBV and HCV infection comparison group. To account for potential confounding by age, sex, and calendar year, we used a frequently matching technique to select the comparison cohort [25],[26]. Therefore, for each participant in the chronic HBV infection alone, chronic HCV infection alone, and chronic HBV/HCV dual infection cohorts, four comparison individuals were randomly selected from the pool of the 935,744 participants without chronic HBV infection, chronic HCV infection, or ARDs. These participants were frequency matched by the year of index date, age (every 10-year span: <20, 20-29, 30-39, 40-49, 50-59, 60-69, 70-79, ≥80 years) and sex at baseline. A total of 199,568 non-chronic HBV and HCV infection participants were finally enrolled in this study (Fig. 1).

### Sociodemographic factors and comorbidities

The sociodemographic factors adjusted for in this study included age, gender, occupation, monthly income, and urbanization of the participant's residential area. Age was grouped into <50 years and ≥50 years. This cutoff was chosen in consideration of the mean age of RA diagnosis in Taiwan which was reported to be 53.7±14.0 years during 2002–2007 [27]. Occupation was divided into blue collar, white collar, and retired and others. The monthly incomes were divided into three levels: ≤NT$15,840, NT$15,841–$25,000, and ≥NT$25,001. An income of NT$15,840 was the government-stipulated minimum wage for full-time employees in Taiwan during the inclusion of the study cohort. Urbanization levels were stratified into three levels, namely urban (levels 1–2), suburban (levels 3–4) and rural (levels 5–7), based on population density [28]. The baseline comorbidities was defined by the Deyo's Charlson Comorbidity Index (CCI) [29].

### Statistical analysis

We first assessed the distribution of sociodemographic factors and comorbidities among participants with chronic HBV infection alone, chronic HCV infection alone, and chronic HBV/HCV dual infections and the comparison participants with non-chronic HBV and HCV infections using Chi-square tests. Multiple comparisons analyses were utilized to determine whether a significant difference existed between pairs of groups [30]. The incidence rates of RA in each study group were measured during the follow-up period. Follow-up time (in person-years) was calculated for each person until RA was diagnosed, or the individual was censored for death or migration, the individual discontinued their enrollment from the NHI, or the follow-up reached the end of 2011. The Kaplan-Meier method was used to estimate the cumulative risk of RA during the 11-year follow-up. The log-rank test was used to evaluate the differences between cohorts. Crude and adjusted hazard ratios (HR) and 95% confidence intervals (CI) for factors associated with the risk of RA were estimated using univariate and multivariate Cox proportional hazards regression models. The adjusted HRs accounted for age, gender, region, occupation, urbanization, income, and Deyo's CCI in the multivariate Cox proportional hazards models. All models were examined for adherence to the proportional-hazards assumption by assessing the log-minus-log survival plots and performing the Schoenfeld test. Results of the log-minus-log survival plots and the Schoenfeld test showed no violations of the proportionality assumption (all *P* values >0.05). Further analyses stratifying for gender and age were also performed.

While patients with HCV infection-related arthritis are generally prescribed non-steroidal anti-inflammatory drugs and low doses of corticosteroids, the course of treatment for patients who have HCV infection and concomitant RA include disease-modifying anti-rheumatic drugs (DMARDs). Most experts recommend starting with nonbiologic DMARDs (methotrexate (MTX)), reserving the use of biologic DMARDs for those who are nonresponsive to nonbiologics (hydroxychloroquine (HCQ) and sulfasalazine (SSZ)) [31]. Hence, we restricted our sensitivity analysis to those RA individuals who were prescribed DMARDs including MTX, HCQ, and SSZ.

The associations between the development of RA and chronic HBV infection with persistent or periodic impairment of liver function were also assessed. Two or more diagnoses of impairment of liver function (ICD-9-CM: 794.8) coded during the follow-up period was defined as persistent or periodic impairment of liver function. All analyses were performed using SAS statistical software for Windows (Version 9.1; SAS Institute, Inc., Cary, NC, USA), and the significance level was set to 0.05.

## Results

### Baseline characteristics and comorbidities of the study subjects

A total of 249,460 participants (140,625 men) with a mean age of 44.7±17.0 years were included in this study. The geographical distribution of participant residence tended to decrease from highest percentage in northern Taiwan (46.0%) to lowest rate in the eastern part of Taiwan and outlaying islands (7.8%). Among the overall population, 54.5%, 33.1% and 12.4% were white-collar, blue-collar, and retired and others respectively. Thirty percent of the participants resided in an urbanized region; on the other hand, 22.3% of their counterparts lived in a rural setting. In terms of monthly income, 22.1% of the participants had incomes higher than NT$25,000 compared with 36.9% who had an income lower than NT$15,840 per month. Only 10.3% of the participants had a score of one or higher on the Deyo's CCI.

The details of the bivariate statistical analyses of sex, age, geographic region, occupation, urbanization level, monthly income, and Deyo's CCI among the chronic hepatitis virus infection cohorts and the non-chronic HBV and HCV infection comparison cohort are presented in Table 1. The participants with chronic HCV infection alone were more likely to be female, older, blue collar workers, living in less urbanized areas and in southern Taiwan, with higher Deyo's CCI scores compared with non-chronic HBV and HCV infection comparison patients. They also had lower incomes than comparison participants. On the contrary, patients with chronic HBV infection tended to be male, younger, and had higher incomes than the comparison individuals. They also had higher Deyo's CCI scores than comparison individuals.

**Table 1:** Baseline characteristics of the chronic hepatitis virus infection cohorts identified in 2000–2010.

	Chronic hepatitis virus infection								
	No^a^ N = 199568		Chronic HBV infection alone N = 35652		Chronic HCV infection alone N = 10253		HBV/HCV infection^b^ N = 3987		
Variable	n	(%)	n	(%)	n	(%)	n	(%)	*P* c
Sex									<0.0001
Women	87068	(43.6)	14936	(41.9)	5084	(49.6)	1747	(43.8)	
Men	112500	(56.4)	20716	(58.1)	5169	(50.4)	2240	(56.2)	
Age, years									<0.0001
<50 years	125684	(63.0)	25577	(71.7)	3823	(37.3)	2021	(50.7)	
≥50 years	73884	(37.0)	10075	(28.3)	6430	(62.7)	1966	(49.3)	
Geographic region									<0.0001
Northern	94520	(47.4)	15959	(44.8)	3093	(30.2)	1285	(32.2)	
Central	39506	(19.8)	7652	(21.5)	2278	(22.2)	836	(21.0)	
Southern	50070	(25.1)	9267	(26.0)	4020	(39.2)	1563	(39.2)	
Eastern and Islands	15467	(7.8)	2774	(7.8)	862	(8.4)	303	(7.6)	
Occupation									<0.0001
White-collar	109526	(54.9)	20734	(58.2)	3972	(38.7)	1780	(44.6)	
Blue-collar	64567	(32.4)	11137	(31.2)	4979	(48.6)	1762	(44.2)	
Retired and others	25475	(12.8)	3781	(10.6)	1302	(12.7)	445	(11.2)	
Urbanization level									<0.0001
Urban	61079	(30.6)	10394	(29.2)	2170	(21.2)	921	(23.1)	
Suburban	95304	(47.8)	17452	(49.0)	4568	(44.6)	1824	(45.7)	
Rural	43170	(21.6)	7804	(21.9)	3514	(34.3)	1242	(31.2)	
Monthly income, NT$									<0.0001
<15,840	75009	(37.6)	12364	(34.7)	3478	(33.9)	1274	(32.0)	
15,841–25,000	80829	(40.5)	14057	(39.4)	5270	(51.4)	1966	(49.3)	
≥25,001	43730	(21.9)	9231	(25.9)	1505	(14.7)	747	(18.7)	
Charlson comorbidity index (CCI)									<0.0001
0	182088	(91.2)	31339	(87.9)	7035	(68.6)	3125	(78.4)	
1	8687	(4.4)	2126	(6.0)	1311	(12.8)	420	(10.5)	
2	5201	(2.6)	1236	(3.5)	822	(8.0)	235	(5.9)	
≥3	3592	(1.8)	951	(2.7)	1085	(10.6)	207	(5.2)	

^a^No: non-chronic HBV and HCV infection comparison cohort.

^b^HBV/HCV infection: chronic HBV and HCV dual infections cohort.

^c^Chi-square test.

### Rheumatoid arthritis between non-chronic HBV and HCV infection and chronic hepatitis virus infection cohorts

**Figure 2:**
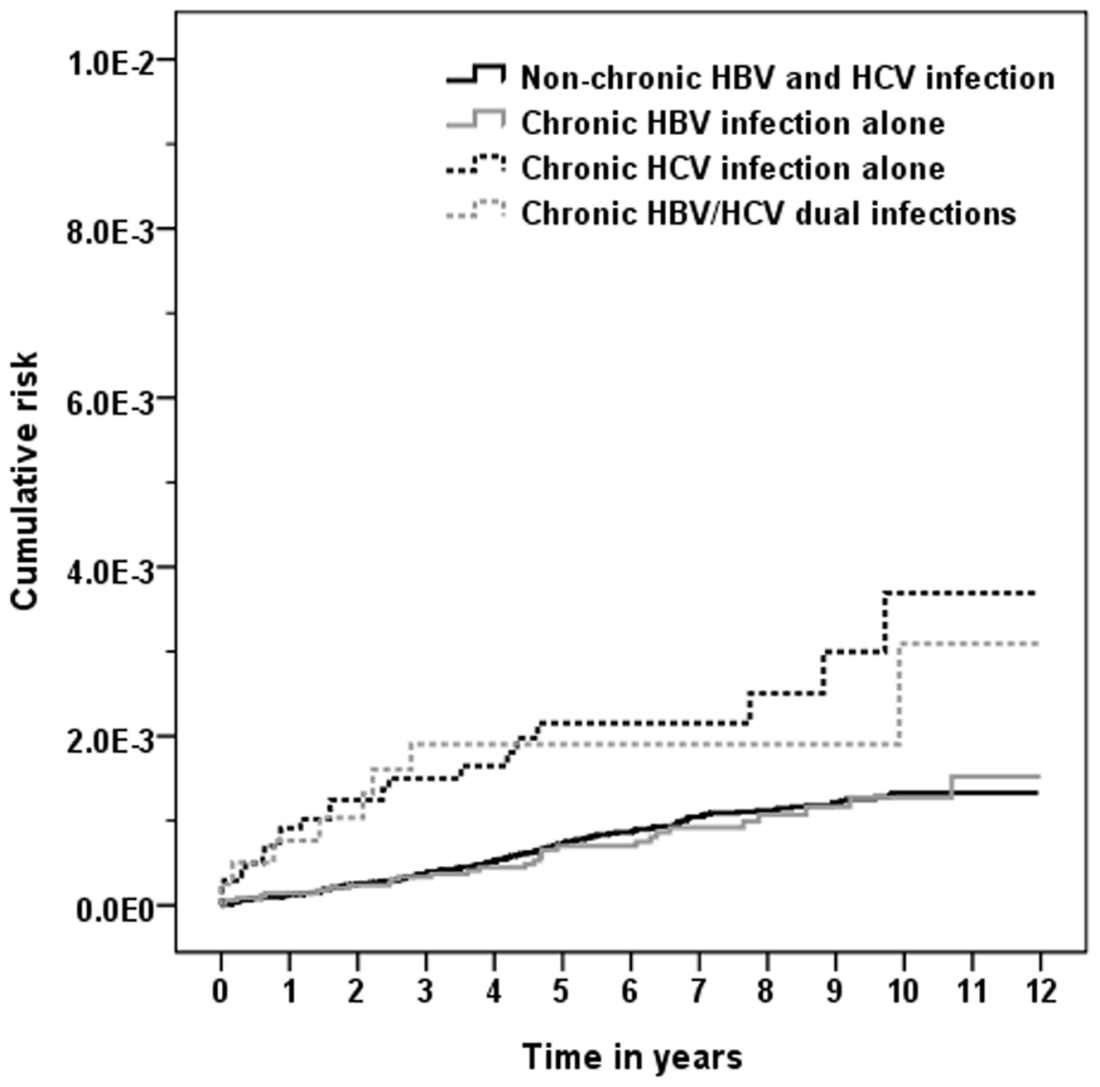
Cumulative risk of rheumatoid arthritis in chronic hepatitis virus infection and non-chronic HBV and HCV infection comparison cohorts over the 11-year follow up period. Log-rank test: Four cohorts, *P*<0.0001; non-chronic HBV and HCV infection comparison cohort vs. chronic HBV infection alone cohort, *P* = 0.834; non-chronic HBV and HCV infection comparison cohort vs. chronic HCV infection alone cohort, *P*<0.0001; non-chronic HBV and HCV infection comparison cohort vs. chronic HBV/HCV dual infections cohort, *P* = 0.023.

As seen in Table 2, 171 (0.09%), 29 (0.08%), 21 (0.20%), and 8 (0.20%) cases of RA were identified among the non-chronic HBV and HCV infection comparison cohort, chronic HBV infection alone cohort, chronic HCV infection alone cohort, and chronic HBV/HCV dual infections cohort, respectively (*P*<.001). The corresponding mean ages of first diagnosis with RA were 52.5±12.3 years, 46.5±13.1 years, 55.1±14.2 years, and 56.5±10.9 years among the four cohorts, respectively. HBV carriers did not appear to be at a significantly higher risk of RA. The incidence of RA was much higher in the cohorts with sole HCV infection and HBV/HCV dual infections than in the non-chronic HBV and HCV infection cohort. The multivariate Cox hazards model showed that the risk of RA was significantly higher among individuals with chronic HCV infection alone than in those in the non-chronic HBV and HCV infection comparison cohort (HR  = 2.03; 95% CI  = 1.27–3.22). The chronic HCV infection alone cohort also had a significantly higher cumulative risk of RA than that of the non-chronic HBV and HCV infection comparison cohort (Fig. 2; log-rank test, the non-chronic HBV and HCV infection comparison cohort vs. the chronic HCV infection alone cohort: *P*<.0001).

**Table 2:** Crude and adjusted hazard ratios for rheumatoid arthritis (RA) associated with chronic hepatitis virus infection.

	Chronic hepatitis virus infection			
	No^b^	Chronic HBV infection alone	Chronic HCV infection alone	HBV/HCV infection^c^
Case of RA	171	29	21	8
Mean age, year	52.5±12.3	46.5±13.1	55.1±14.2	56.5±10.9
Person-year	1116275	197492	48416	23379
Incidence, per 10,000 person-years	1.53	1.47	4.34	3.42
Crude HR (95% CI)	1.00(ref)	0.96(0.65–1.42)	2.84(1.80–4.46)‡	2.23(1.10–4.53)*
Adjusted HR (95% CI)^a^	1.00(ref)	1.09(0.74–1.63)	2.03(1.27–3.22)†	1.92(0.94–3.92)

^a^Adjusted for sex, age, region, occupation, urbanization, income, and Charlson comorbidity index (CCI).

^b^No: non- chronic HBV and HCV infection comparison cohort.

^c^HBV/HCV infection: chronic HBV and HCV dual infections cohort.

*p<0.05,

† p<0.01,

‡ p<0.0001.

### Adjusted HRs of rheumatoid arthritis with chronic HBV and HCV infections by sex and age

Table 3 shows the gender- and age-specific HRs of RA for the chronic HBV infection alone, chronic HCV infection alone, and chronic HBV/HCV dual infections cohorts compared to the non-chronic HBV and HCV infection comparison cohort. The gender-specific HRs of RA associated with chronic HCV infection alone were 1.88 (95% CI  = 1.08–3.25) for women and 2.57 (95% CI  = 1.08–6.13) for men (P-interaction  = 0.493). The HRs for RA among participants infected with chronic HCV infection alone were significantly higher among those aged ≥50 years old (HR  = 1.83, 95% CI  = 1.05–3.19) (P-interaction  = 0.591). The increased risk for RA was significant in females with chronic HBV/HCV dual infections (HR  = 2.27, 95% CI  = 1.06–4.89); however P-interaction (p = 0.386) remained insignificant.

**Table 3:** Adjusted hazard ratios for rheumatoid arthritis (RA) associated with chronic hepatitis virus infection by sex and age.

			No^d^			Chronic HBV infection alone			Chronic HCV infection alone			HBV/HCV infection^f^
RA^a^	Cases	I^e^	HR (95% CI)	Cases	I^e^	HR (95% CI)	Cases	I^e^	HR (95% CI)	Cases	I^e^	HR (95% CI)
Sex^b^												
Female	119	2.44	1.00(ref)	24	2.86	1.32(0.85–2.06)	15	6.09	1.88(1.08–3.25)*	7	6.58	2.27(1.06–4.89)*
Male	52	0.83	1.00(ref)	5	0.44	0.59(0.24–1.49)	6	2.52	2.57(1.08–6.13)*	1	0.78	0.92(0.13–6.69)
Age, years^c^												
<50	73	0.97	1.00(ref)	19	1.25	1.34(0.81–2.22)	6	2.85	2.29(0.98–5.35)	2	1.54	1.35(0.33–5.52)
≥50	98	2.67	1.00(ref)	10	2.21	0.81(0.42–1.55)	15	5.48	1.83(1.05–3.19)*	6	5.79	2.05(0.89–4.69)

^a^All *P* values for interaction between chronic hepatitis virus infection and age or sex were >0.05.

^b^Adjusted for age, region, occupation, urbanization, income, and Charlson comorbidity index (CCI).

^c^Adjusted for sex, region, occupation, urbanization, income, and Charlson comorbidity index (CCI).

^d^No: non- chronic HBV and HCV infection comparison cohort.

^e^Incidence: per 10,000 person-years.

^f^HBV/HCV infection: chronic HBV and HCV dual infections cohort.

*p<0.05.

### Adjusted HRs for rheumatoid arthritis among patients with chronic HBV and HCV infections and having been prescribed disease-modifying anti-rheumatic drugs (DMARDs) by sex and age

Table 4 presents an analysis conducted after restricting the study cohort to those participants who received a prescription for DMARDs (HCQ, SSZ, or MTX). The risk of RA was still significantly higher among subjects with chronic HCV infection alone than among those in the non-chronic HBV and HCV infection comparison cohort (HR  = 1.89; 95% CI  = 1.15–3.11). The gender-specific HR for RA associated with chronic HCV infection alone was 2.30 (95% CI  = 1.24–7.23) for men, while the association did not reach the statistical level among women.

**Table 4:** Adjusted hazard ratios for rheumatoid arthritis (RA) among subjects with chronic hepatitis virus infection and prescribed disease-modifying anti-rheumatic drugs (DMARDs) by sex and age.

	No^d^	Chronic HBV infection alone	Chronic HCV infection alone	HBV/HCV infection^e^
	HR (95% CI)	HR (95% CI)	HR (95% CI)	HR (95% CI)
All^a^	1.00(ref)	1.15(0.77–1.73)	1.89(1.15–3.11)*	1.85(0.86–3.95)
				
Sex^b^				
Female	1.00(ref)	1.34(0.85–2.10)	1.60(0.87–2.93)	2.08(0.91–4.76)
Male	1.00(ref)	0.70(0.28–1.76)	2.30(1.24–7.23)*	1.09(0.15–7.94)
				
Age, years^c^				
<50	1.00(ref)	1.36(0.81–2.29)	2.03(0.81–5.12)	1.44(0.35–5.91)
≥50	1.00(ref)	0.89(0.46–1.71)	1.75(0.97–3.18)	1.90(0.77–4.69)

^a^Adjusted for sex, age, region, occupation, urbanization, income, and Charlson comorbidity index (CCI).

^b^Adjusted for age, region, occupation, urbanization, income, and Charlson comorbidity index (CCI).

^c^Adjusted for sex, region, occupation, urbanization, income, and Charlson comorbidity index (CCI).

^d^No: non-chronic HBV and HCV infection comparison cohort.

^e^HBV/HCV infection: chronic HBV and HCV dual infections cohort.

*p<0.05.

### Associations between the development of rheumatoid arthritis and chronic HBV infection with impairment of liver function

Table 5 shows the HRs for RA among individuals with chronic HBV infection alone with persistent or periodic impairment of liver function compared to the non-chronic HBV infection without impairment of liver function comparison cohort. The HRs of RA associated with chronic HBV infection were 2.64 (95% CI  = 0.84–8.28) with persistent or periodic impairment of liver function and 1.05 (95% CI  = 0.70–1.60) without persistent or periodic impairment of liver function.

**Table 5:** Association between the development of rheumatoid arthritis (RA) and chronic HBV infection concurrent with persistent or periodic impairment of liver function.

Persistent or periodic impairment of liver function	No^a^			Chronic HBV infection alone		
	Cases	I^b^	HR (95% CI)^c^	Cases	I^b^	HR (95% CI)^c^
No	166	1.51	1.00(ref)	26	1.37	1.05(0.70–1.60)
Yes	5	3.13	1.85(0.76–4.52)	3	3.75	2.64(0.84–8.28)

^a^No: non-chronic HBV and HCV infection comparison cohort.

^b^Incidence: per 10,000 person-years.

^c^Adjusted for sex, age, region, occupation, urbanization, income, and Charlson comorbidity index (CCI).

## Discussion

In this study, patients with chronic HCV infection but not chronic HBV infection were at a higher risk for subsequently developing RA. These findings suggest that chronic HCV infection may be a rare but significant precipitating factor of RA.

Many studies have attempted to clarify the association between rheumatic diseases (especially RA) and chronic HCV infection. The prevalence of chronic HCV infection among patients with rheumatic diseases has been reported to vary across study sites, which may reflect the background infection rates of different countries. Some studies from Brazil and Spain reported higher HCV seropositive rates in patients with rheumatic diseases [12],[13], while others failed to reveal a difference from the background prevalence rates of the region under study [32]–[34]. Palazzi and his colleagues estimated that HCV-related arthritis could affect up to 4% of subjects suffering from chronic HCV infection [35]. In a large cohort of HCV infected patients, this study only detected an RA prevalence of 0.22%, making it unlikely that the HCV infection selection criteria utilized by this study targeted HCV-related arthritis.

Clinically and serologically, HCV-related arthropathy is hard to be distinguished from an initial presentation of true RA [31],[36],[37], with many patients with RA-like HCV-related polyarthritis fulfilling the American College of Rheumatology (ACR) criteria for RA [38]. Antibodies against cyclic citrullinated peptide (Anti-CCP) are more specific than RF in the diagnosis of RA [36],[39], however false positives are still a concern for patients with HCV infection [40]. Therefore, a correct diagnosis of RA in HCV-infected patients relies on the judicious clinical evaluation of synovial hyperplasia, bone erosion, and treatment response. Our findings are strengthened by our analysis conducted after restricting our RA subjects to patients who received a prescription of DMARDs. Thus, our results support the notion that chronic HCV infection is associated with the development of RA.

Some previous studies have suggested that chronic HBV infection may act as a trigger for the development of ARDs [14],[15]. Higher positive rates of RF among asymptomatic HBV carriers than controls have also been reported [16],[17]. However, in an investigation of HBV serological markers in 239 rheumatic patients, Permin et al. found no relationship between HBV infection and most rheumatic diseases, with the exception of polyarteritis nodosa [19]. Lee et al. also reported that 20–75% of patients with chronic HBV infection may be positive for RF, but usually do not develop or suffer from RA despite presenting with arthralgia or arthritis [18]. Our results garnished from a population-based study conducted in a high HBV infection endemic area support the findings of prior studies which reported that there is most likely no association between chronic HBV infection and RA.

Even though HBV and HCV are both chronic hepatitis viruses and are associated with higher seropositive rates of RF, it is of interest why they engender different RA risks. Most extrahepatic manifestations of HCV infection are secondary to the elicitation of autoimmune reactions, generalized deposition of immune complexes, or lymphoproliferative disorders. Some authors suggest only HCV, not HBV, has the lymphotrophic character that is suspected to be the cause of some HCV-associated extrahepatic manifestations, which provides a possible theoretical explanation for our findings [41].

This study had several limitations. First, selection bias might arise from the use of the sampled NHI data of 1,000,000 insurants as it does not cover the entire population of Taiwan. In Taiwan, NHI is a single-payer compulsory social insurance plan established in 1995 which centralizes the disbursement of health-care funds and provides equal access to health care for all citizens. By the end of 2004 the success of this program was evident with approximately 99% of Taiwan's 23 million population having been enrolled [24]. The high coverage rate of the NHI makes this program's corresponding administrative data very representative of the overall Taiwanese population.

Second, the definition of RA cases was dependent on ICD-9-CM codes and their catastrophic disease registration, and therefore may be of concern. Clinicians make diagnoses according to not only serological tests but also based on evidence of synovial hyperplasia and bone erosion. Therefore, the RA prevalence among patients with chronic HCV infection in this study (0.20%) was much lower than the RF positive rate in patients with chronic HCV infection reported by a prior study in Taiwan (18% according to Hu, et al) [42].

Third, some patients with chronic hepatitis virus infections do not have obvious clinical symptoms and, therefore, might not seek medical attention. As a result, some patients with asymptomatic hepatitis virus infections were included in the comparison cohort. However, if chronic HBV or HCV infection is associated causally with RA, this misclassification would bias the results toward the null and underestimate the risk. Thus, the results of this study represent a conservative estimate.

Fourth, in this secondary data analysis, we were not able to illustrate the risk of RA in participants with chronic HBV infection and abnormal liver function defined by serum Alanine Aminotransferase (ALT) level. Instead, we defined the impairment of liver function based on ICD-9-CM coding. Hence, we are not able to comment on any potential dose-response relationship between liver function (ALT) and RA risk among chronic HBV carriers. Further studies will be needed to better explore this association.

Fifth, some RA risk factors, such as family history, smoking, diet, and hormone levels are unavailable in the insurance claims database [43]. Therefore, we can't rule out some of the potential confounding effects associated with these factors. Fifth, the majority of the residents in Taiwan are of Chinese Han ethnicity. Hence, whether our results hold in other ethnic groups awaits further study.

## Conclusions

In this nationwide population-based cohort study conducted in a country where both HBV and HCV infections are highly prevalent, we found individuals with chronic HCV infection to be at a significantly higher risk of RA. This study also contributes to the body of evidence suggesting that chronic HBV infection is most likely not associated with an increased risk of RA. Physicians treating patients with chronic HCV infection presenting with arthralgia or arthritis should be aware of their increased risk of RA. Viral infection has long been proposed to be a trigger of autoimmune disease and our study may provide a model of gene-environment interaction between chronic HCV infection and RA development.
