# Correction: Chronic Hepatitis C Virus Infection Is Associated with the Development of Rheumatoid Arthritis: A Nationwide Population-Based Study in Taiwan

**DOI:** 10.1371/journal.pone.0166508

**Published:** 2016-11-09

**Authors:** Fu-Hsiung Su, Chien-Sheng Wu, Fung-Chang Sung, Shih-Ni Chang, Chien-Tien Su, Ying-Hua Shieh, Chih-Ching Yeh

There is an error in affiliation 2 for authors Fu-Hsiung Su and Ying-Hua Shieh. Affiliation 2 should be: Department of Family Medicine, School of Medicine, College of Medicine, Taipei Medical University, Taipei, Taiwan.
